# Fatal Errors

**Published:** 1882-05

**Authors:** Ad. Lippe

**Affiliations:** Philadelphia


### FATAL ERRORS.

Ad. Lippe, M. D.

It is a fatal error for a homœopathist to teach that syphilis is a surgical disease. It is also a fatal error to teach that the single remedy and the minimum dose are unsettled questions. It is worse than a fatal error to teach that the local application of acid nitrate of Mercury will arrest and cure a case of neglected chancre eating the penis off. And it may please the reader to learn that all these huge fatal errors, and some minor ones, were committed by our friend, the learned editor of the *United States Medical Investigator*, Feb. 15th, 1882, page 206, in just sixteen lines of a *classical* paragraph.

What constitutes a surgical disease? The consequences of mechanical injuries are surgical diseases, and must receive the needful mechanical treatment. After mature experience the famous Dr. Ricord discards as injurious all local treatment of venereal diseases, and advises constitutional treatment. Is the very learned editor of the *United States Medical Investigator* playing hide and seek with the allopathic fraternity, who sneak around the partition wall into our position to find the editor of one journal with his following just sneaking around the other side of the partition wall to advocate then and there the abandoned teachings of the fossils, the regulars, in their abandoned camp. This seems to be the order of the day. We see the nursery play "puss in the corner" performed by a liberal but playful set of men. Just as fast as the allopathists abandon their old errors and accept Hahnemann's teachings, do the eclectics, sailing under the homœopathic colors, pick up the ancient errors and insist upon grafting them on Homœopathy. The single remedy and the minimum dose these philosophers erroneously say are unsettled questions. Are they? And if you disbelieved their correctness, why did you join our ranks? The allopathists are advocating just now exactly what these eclectics doubt! Will the learned editor read a February number of the *New York Medical Record*, professedly an allopathic journal? It proclaims the law of the Similars the *only* law of cure. The eclectics in that respect contradict liberally both the homœopathicians and allopaths. Where are they, and where are they going to? Organize themselves under the freedom-tree we sincerely hope.

